# Breaking the Rebellion: Photodynamic Inactivation against *Erwinia amylovora* Resistant to Streptomycin

**DOI:** 10.3390/antibiotics11050544

**Published:** 2022-04-19

**Authors:** Annette Wimmer, Michael Glueck, Wenzi Ckurshumova, Jun Liu, Michael Fefer, Kristjan Plaetzer

**Affiliations:** 1 Laboratory of Photodynamic Inactivation of Microorganisms, Department of Biosciences and Medical Biology, Paris Lodron University of Salzburg, Hellbrunnerstr. 34, 5020 Salzburg, Austria; annette.wimmer@plus.ac.at (A.W.); michaeljohannes.glueck@plus.ac.at (M.G.); 2 Suncor AgroScience, 2489 North Sheridan Way, Mississauga, ON L5K 1A8, Canada; wckurshumova@suncor.com (W.C.); juliu@suncor.com (J.L.); mfefer@suncor.com (M.F.)

**Keywords:** Photodynamic Inactivation, antimicrobial resistance, plant disease, *Erwinia amylovora*, fire blight, natural compounds, chlorophyllin, streptomycin

## Abstract

Global crop production depends on strategies to counteract the ever-increasing spread of plant pathogens. Antibiotics are often used for large-scale treatments. As a result, *Erwinia amylovora*, causal agent of the contagious fire blight disease, has already evolved resistance to streptomycin (Sm). Photodynamic Inactivation (PDI) of microorganisms has been introduced as innovative method for plant protection. The aim of this study is to demonstrate that *E. amylovora* resistant to Sm (*E. amylovora* ^SmR^) can be killed by PDI. Two photosensitizers, the synthetic B17-0024, and the natural derived anionic sodium magnesium chlorophyllin (Chl) with cell-wall-permeabilizing agents are compared in terms of their photo-killing efficiency in liquid culture with or without 100 µg/mL Sm. In vitro experiments were performed at photosensitizer concentrations of 1, 10 or 100 µM and 5 or 30 min incubation in the dark, followed by illumination at 395 nm (radiant exposure 26.6 J/cm^2^). The highest inactivation of seven log steps was achieved at 100 µM B17-0024 after 30 min incubation. Shorter incubation (5 min), likely to represent field conditions, reduced the photo-killing to 5 log steps. Chlorophyllin at 100 µM in combination with 1.2% polyaspartic acid (PASA) reduced the number of bacteria by 6 log steps. While PASA itself caused some light independent toxicity, an antibacterial effect (3 log reduction) was achieved only in combination with Chl, even at concentrations as low as 10 µM. Addition of 100 µg/mL Sm to media did not significantly increase the efficacy of the photodynamic treatment. This study proves principle that PDI can be used to treat plant diseases even if causative bacteria are resistant to conventional treatment. Therefore, PDI based on natural photosensitizers might represent an eco-friendly treatment strategy especially in organic farming.

## 1. Introduction

By international law, countries have an obligation to ensure that everyone receives adequate, in quantity and quality, and healthy food, as stated in Article 11 of the International Covenant on Economic, Social and Cultural Rights. This is also supported by the goals of the 2030 Agenda of Sustainable Development of the United Nations [1]. The crop production sector plays a key role in global food security. The linchpin is the realization of this human right, as global crop production has to prepare for major challenges in the future. Continued population growth is almost inevitable. By the year 2030, the world population is projected to increase to around 8.5 billion, by 2050 to between 9.4 and 10 billion and by 2100 to between 10 and 12.5 billion [2]. Moreover, the demand for per capita food consumption, i.e., the dietary energy measured in kcals per capita per day will steadily increase on a worldwide basis [3]. Both these facts enhance the need of raising food production levels. However, it is a matter of fact that global land allocated to arable production or permanent crops will not expand accordingly; rather, it is projected that the area for crop production will remain constant. Ensuring higher food supply, the remaining cropland area has to be managed in a way to extract higher harvest yield per hectare. Crop production systems must be transformed to be more efficient, a consequence that enhances the spread of plant pathogens.

Phytopathogens cause great economic and social losses. Among crops, the total global potential loss due to pests is over 50%, with plant pathogens causing an estimated 16% [4]. The ever-increasing threat from plant pathogens inevitably leads to high inputs of fertilizers and pesticides due to the current lack of sustainable alternative methods. Pathogens and their harmful effects are of particular relevance when perennial crops are involved. To name an example, the phytopathogen *Erwinia amylovora* (Burrill 1882) Winslow et al. 1920, the causal agent of the contagious fire blight disease, infects many cultivated plants such as apples and pears and some other members of the Rosaceae family [5]. Fire blight can destroy an entire orchard in one growing season. Moreover, a single severe outbreak can disrupt orchard production for years. This was illustrated by the epidemic outbreak of fire blight in Southwest Michigan in 2000, when some 400,000 apple trees had to be removed and the economic loss amounted to over USD 42 million [6]. *Erwinia amylovora* is capable of infecting different plant organs, such as blossoms, fruits, vegetative shoots, woody tissues, and rootstock crowns [7]. Characterized by a disease cycle, fire blight includes several distinct phases of disease including blossom blight, shoot blight, and rootstock blight. In spring, especially in warm and humid weather conditions, the pathogen can spread rapidly by wind, water, or insects infecting blossoms of plants by transfer of contaminated pollen. After inoculation of surfaces, bacteria enter through nectaries, lenticels, and stomata. This leads to infections of shoots and water vessels are blocked until blossom blight. The only effective way to prevent infection is in a very early stage of the disease cycle. If growers fail to control blossom blight, shoot blight and rootstock blight will inevitably develop during summer and fruits can be infected. Bacteria hibernate in cankers, and in spring they are disseminated from canker to blossom by insects or rain and the disease cycle closes. To combat fire blight, growers use chemical, biological, and cultural controls [5]. Chemical control of the disease can be achieved by application of copper-based compounds to blossoms, but the toxicity towards *Erwinia* is limited and negative effects of copper to soil and ground water are content of a controversial discussion. Still, the most effective way to control fire blight is the use of antibiotics [7].

The antibiotic streptomycin (Sm) is used to treat fire blight in the field in many countries posing an ongoing threat of evolving resistance. Resistant strains of *E. amylovora* are already common in orchards of the western United States and British Columbia in Canada, and in turn lead to high economic losses [8]. In general, there are two phenotypes of streptomycin resistance detected in strains of *E. amylovora*: a moderately resistant one, having a streptomycin minimum inhibitory concentration (MIC) of 500 to 750 µg/mL; and a highly resistant one, with an MIC of 2 mg/mL [8]. Moderate resistance against streptomycin is associated with acquisition of the *strA-strB* gene pair [9]. These two linked genes can be acquired by transposons or plasmids and both encode for phosphotransferase enzymes that modify streptomycin to a non-toxic form [10]. The other mechanism for streptomycin resistance, which induces tolerance to high amounts, is based on a spontaneous point mutation of the chromosomal *rpsL* gene [9]. More specifically, it is a mutation at codon 43, where lysine is replaced by an arginine in most cases. Other resistant strains of *E. amylovora* exist, in which lysine is changed to asparagine or threonine, suggesting that resistance has arisen independently and been selected for multiple times. This *rpsL* gene is encoding for the S12 protein of the 30S small ribosomal subunit, the target of streptomycin. In consequence, streptomycin cannot bind at the 30S subunit and thus, the ribosome cycle of protein synthesis can still proceed well [8].

In the European Union and Brazil, the use of streptomycin in crop protection has been banned due to the rapid evolution of the resistance of *E. amylovora* to this antibiotic [11]. As a consequence, one of the main tasks for plant protection management will be the establishment of sustainable best practices in preventing both plant diseases as well as evolving antimicrobial resistance of microorganisms. This goal is reflected by the 2030 Agenda of Sustainable Development of the United Nations [1] and results in an urgent need for effective, eco-friendly and environmentally safe as well as non-phytotoxic treatment methods, that can be recommended for satisfactory and reliable application in the field.

Photodynamic Inactivation (PDI) of microorganisms has been introduced as an alternative method to complement or replace currently used plant protection treatments [12,13]. PDI is based on the principle that a per se non-toxic photosensitive substance (photosensitizer, PS) is activated by visible light, forming reactive oxygen species (ROS), which in turn directly induce photo-killing in target cells [14]. Important processes include the oxidation of various biomolecules such as membrane lipids, amino acids and/or nucleic acids, disturbing the normal function of the target pathogen and ultimately killing the cell [15]. Due to its universal mechanism of action PDI can kill all types of pathogenic microorganisms, including bacteria, fungi, parasites and viruses [16]. The potential of PDI was already revealed at the beginning of the 20th century [17,18,19]. However, with the discovery of penicillin and the development of further antibiotics in the following years it was almost forgotten. PDI represents an alternative technique in which the action mechanism is fundamentally different from the key–lock principle of conventional antibiotics. The PDI mechanism is non-specific, offering a random oxidation of target compounds by ROS to prevent the evolution of bacterial resistance mechanisms to cope with drugs such as antibiotics [16]. The development of resistance to PDI treatment is almost excluded due to its swift and multi-target mode of action.

Photosensitizers play the key role in PDI treatments as generators of the actual active product (ROS). The group of natural PS stands out due to their high biocompatibility. They are ideally non-toxic to the environment as well as available in large quantities and at a low price. Most of them are extracted from plants. For example, chlorophyllins are porphyrins derived from chlorophyll. A major source is the cyanobacterium (*Spirulina platensis*), but it can also be extracted from green plant products, such as spinach (*Spinacia oleracea*). The naturally derived, semisynthetic anionic PS sodium magnesium chlorophyllin (Chl) has been previously used in PDI against plant pathogens [12,13]. Due to its absorption properties, it can be photo-activated in the blue (~410 nm) or red (~630–660 nm) wavelength range [20]. Modifications of photosensitizers (i.e., by cationic moieties) often result in enhanced efficacy. For example B17-0024, a mixture of two chlorin e6 derivatives with cationic moieties at physiological pH, has recently been shown to be 10 times more effective against plant bacteria and fungi compared to anionic Chl [12,13].

The aim of this study was to test whether a streptomycin-resistant strain of *E. amylovora* (*E. amylovora* ^SmR^) can be killed by PDI using natural substances as photoactive compounds at the same efficiency as the susceptible strain. This work expands on the previously reported success of PDI against the wild-type strain of *E. amylovora* (*E. amylovora* *^WT^*) [12]. For a low eco-toxicological impact, we use the naturally derived photosensitizer Chl, registered as a food additive (E140). Chlorophyllin was combined with EDTA disodium salt dihydrate (Na_2_EDTA) and polyaspartic acid (PASA) for cell wall permeabilization of Gram (-) *Erwinia*. The photo-killing efficiency was compared to the synthetic PS B17-0024 without additives. To investigate the possible additive or synergistic effects of Sm, experiments were performed in presence or absence of Sm in the media.

## 2. Results and Discussion

Resistance to antibiotics is increasing at an alarming speed in all application fields, including agriculture. Infection of annual crops may result in a complete loss with dramatic consequences for the farmer. The situation is even worse for plants which need to grow for several years before developing fruits, such as apple and pear trees. The latter accounts for *E. amylovora*, the bacterial pathogen causing fire blight disease in members of the Rosaceae family. Up to date, different types of resistant strains of *E. amylovora* have caused severe crop losses in the United States and in Canada [21]. The increasing resistance to antibiotics is a serious problem in two aspects: on the one hand, there is a lack of effective treatments against resistant pathogens in plant production; and on the other hand, there is increasing evidence that resistance genes can be exchanged between free-living bacteria and human pathogens via horizontal gene transfer [22]. European Union policy has already recognized the urgent need for action and decreed a general ban on plant protection products containing streptomycin within the EU [23]. Although this helps to fight back resistance, it leaves growers with no other treatment option than to cut down infected trees. PDI of microorganisms has recently been introduced and validated against bacterial and fungal plant pathogens [12,13]. Whether this also extends to resistant plant pathogens, such as the Sm-resistant strain of *E. amylovora*, has not yet been proven.

### 2.1. Growth Analysis of *Erwinia amylovora*

**Figure 1:**
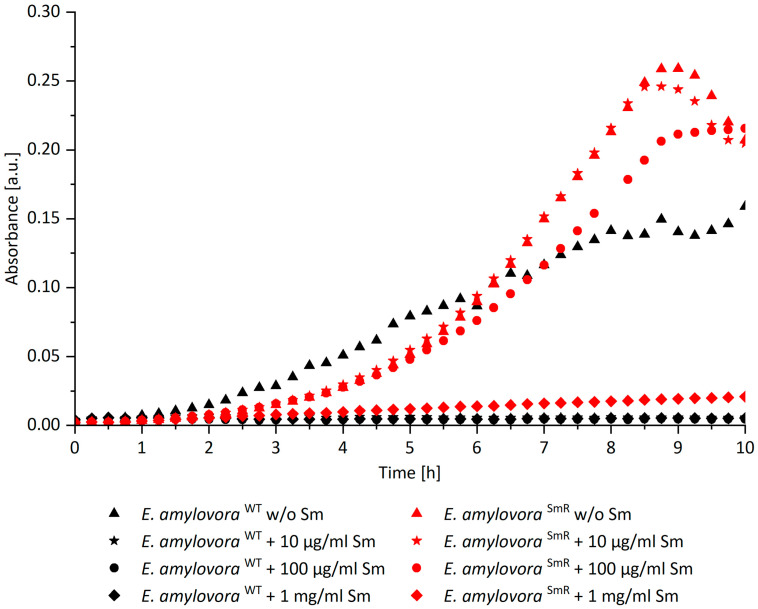
Growth curves of *E. amylovora* ^SmR^ (red) and *E. amylovora* ^WT^ (black) in MPM broth supplemented with different concentrations of Sm. The graph shows light absorbance at 600 nm corrected by blank (MPM) over 10 h. The growth of *E. amylovora* ^SmR^ was unaffected in MPM with an Sm concentration of maximal 100 µg/mL. *E. amylovora* ^WT^ showed no growth at any concentration of Sm.

To identify the limit of tolerance of *E. amylovora* ^SmR^ against Sm, automatic growth curves were recorded. *E. amylovora* ^SmR^ shows unaffected growth in MPM broth supplemented with Sm up to 100 µg/mL and proliferation was abolished at 1 mg/mL (Figure 1). McManus et al. reported resistance of *E. amylovora* ^SmR^ in lysogeny broth up to 2 mg/mL [8], which we were able to confirm when using this medium (data not shown). However, to establish comparability with our previous study [12], bacteria were cultured in MPM throughout this study. As expected, *E. amylovora* ^WT^ showed no growth when Sm was added. For PDI experiments with *E. amylovora* ^SmR^ 100 µg/mL Sm was added when indicated.

### 2.2. Photodynamic Inactivation of E. amylovora ^SmR^ Based on B17-0024 without Streptomycin

**Figure 2:**
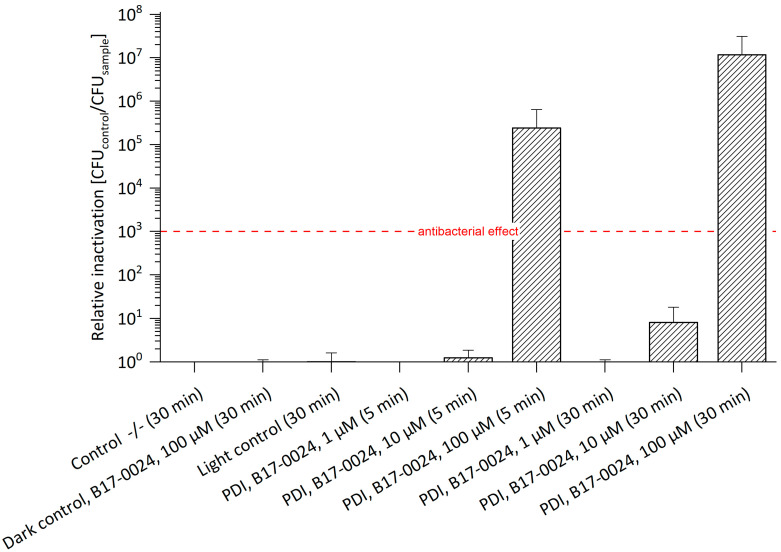
Relative Inactivation of *E. amylovora* ^SmR^ using B17-0024 at 1 μM, 10 μM or 100 μM (*n* = 3). Bacteria cultured/plated on MPM without Sm. Incubation periods (in brackets) were 5 min or 30 min. Illumination was done at 395 nm and 26.6 J/cm^2^. Number of CFUs of Co-/- (mean): 4.6 × 10^7^. The maximal relative inactivation of 7 log steps was achieved at a concentration of 100 µM and an incubation period of 30 min. An antibacterial effect of 5 log steps was observed with 100 µM B17-0024 even after 5 min incubation. The red dashed line corresponds to a reduction of 3 log units. Control -/-: double negative control.

For testing the potential of PDI, a first set of experiments against *E. amylovora* ^SmR^ was performed with B17-0024. Photo-activation of 100 µM B17-0024 resulted in an antibacterial effect against *E. amylovora* ^SmR^ with a relative inactivation of 2.4 × 10^5^ with 5 min incubation and of 1.2 × 10^7^ with 30 min incubation (Figure 2, Table S1). Compared to *E. amylovora* ^WT^, 100 µM B17-0024 led to an antibacterial effect of four log steps after 5 min, and of six log steps after 30 min incubation. B17-0024 in concentrations of 1 µM and 10 µM did not result in a photo-bactericidal effect irrespective of the incubation period. Again, this is in line with previous studies [12].

In general, cationic PS are required for a successful PDI against Gram (-) bacteria [24]. Jesus et al. [25] and Martin et al. [26] reported successful PDI treatments with cationic porphyrins against Gram (-) *Pseudomonas syringae* pv. *actinidiae*, which affects kiwi plants. In vitro photo-activation based on 5 µM of 5, 10, 15, 20-tetrakis(1-methylpyridinium-4-yl)porphyrin tetraiodide (Tetra-Py + -Me) with 10 min incubation in the dark and 60 min illumination under artificial PAR white light at an irradiance of 4.0 mW/cm^2^ resulted in a decrease of viable bacterial cells of 6 log steps. A reduction of 3 log steps was observed after just 5 min of illumination [25]. Martins et al. confirmed these data: in vitro photo-activation of 5 µM of a mixture of different cationic PS after 10 min incubation in the dark and 10 min illumination with artificial white light (380–700 nm) at 4.0 mW/cm^2^ led to a reduction of bacterial count of 3 log steps, and after 60 min illumination to a reduction of 7 log steps [26]. Another study by Lopes and coworkers evaluated the effectiveness of PDI based on cationic phenothiazines in combination with potassium iodide against *Pseudomonas syringae* pv. *actinidiae* on kiwifruit pollen. A reduction of bacteria by 8 log steps was achieved when using 5 µM New Methylene Blue combined with 100 mM potassium iodide (100 mM) as potentiator after 90 min of illumination using a LED projector as light source. The illumination period could even be halved to 45 min when using 5 µM Methylene Blue supplemented with potassium iodide, resulting in a comparable antibacterial effect. Most interestingly, photo-treatment had no negative effects on pollen germination [27], thus demonstrating the applicability of the approach. Compared to our work, these studies used antibiotic-susceptible plant pathogens and reported that PDI is effective even with lower concentrations of PS as well as at a very low light intensity, which is about 1/10 lower than the one employed in this study. However, for the present study, light intensity should not be a limiting factor and was therefore kept sufficiently high.

### 2.3. Photodynamic Inactivation of E. amylovora ^SmR^ Based on B17-0024 Supplemented with Streptomycin

**Figure 3:**
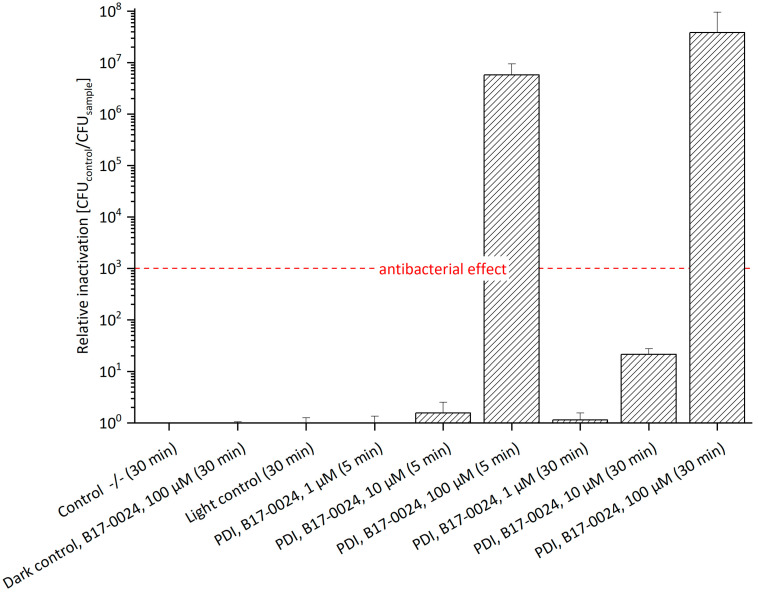
Relative Inactivation of *E. amylovora* ^SmR^ using B17-0024 at 1 μM, 10 μM or 100 μM concentration (*n* = 3). Bacteria cultured/plated on MPM with 100 µg/mL Sm. Incubation periods (in brackets) were 5 min or 30 min. Illumination was done at 395 nm and 26.6 J/cm^2^. Number of CFU of Control -/- (mean): 7.1 × 10^7^. With 100 µM B17-0024 a relative inactivation of 6 log steps after only 5 min incubation and 7 log steps after 30 min can be achieved.

The effects and mechanisms of action of PDI in combination with antibiotics have been reported previously [28,29,30,31,32]. To examine the effect of Sm in combination with the PS, we supplemented 100 µg/mL Sm to the bacterial culture and agar plates. The presence of Sm slightly increased the efficacy of photo-treatment (Figure 3, Table S2). At a concentration of 100 µM B17-0024 a relative inactivation of 2.9 × 10^6^ was achieved after 5 min incubation. Thus, the antibacterial effect was 1 log unit higher than without Sm (see Figure 2). After 30 min incubation, 100 µM B17-0024 led to a relative inactivation of 3.8 × 10^7^, which is slightly higher than without Sm (1.2 × 10^7^). As working hypothesis for these minor effects addition of Sm could pose increased stress to bacterial cells that suffer from photo-damage but activate repair mechanisms to survive. Sm probably affects these repair functions, such as an impaired protein synthesis leading to a limited repair capability of photo-damage as Sm disrupts the polyribosome metabolism respectively protein synthesis in bacteria [33]. However, it can be assumed that Sm inhibits other proteins that may affect the structure and function of the cell. A previous study by Perales-Vela et al. [34] reported that, in some micro algae, exposure to UV light with the presence of Sm inhibited not only the protein synthesis but also the repair processes of these proteins. Another possibility could be oriented towards the concept of collateral sensitivity, in which the evolution of resistance to antibiotics results in increased sensitivity to another antimicrobial agent that has a completely different mechanism of action [35,36]. Hence, it might be a hint that *E. amylovora* ^SmR^ reacts with a slight increased sensitivity to a combination of PDI and Sm. However, Sm supplement did not significantly enhance the efficacy of PDI.

The results presented in this study prove that *E. amylovora* ^SmR^ can be successfully photo-killed by B17-0024. However, for application in orchards very large quantities of this substance are needed. Typically, 2000 L of water per hectare are sprayed on a field. An effective concentration of 100 µM B17-00024 corresponds to 147.4 g PS per 2000 L and hectare. Natural PS are relatively inexpensive and therefore allow for economically feasible treatments. Many of these compounds can be extracted from plants. Examples are hypericin (extracted from St. John’s wort), curcumin (from turmeric) and chlorophyllin (from cyanobacterium or extracted from green plants).

### 2.4. Photodynamic Inactivation of E. amylovora ^SmR^ Based on Na-Mg-Chlorophyllin Supplemented with Cell Wall Permeabilizing Additives

Na-Mg-Chlorophyllin is approved as food additive E140 and is therefore highly biocompatible as well as available in large quantities. As an anionic molecule, Chl cannot cross the Gram (-) cell wall due to the presence of negatively charged lipopolysaccharides of the outer membrane [37]. The combination of Chl with cell wall permeabilizing agents has been shown to overcome this limitation [12]. Preliminary experiments on PDI using Chl supplemented with 5 mM Na_2_EDTA against *E. amylovora* ^SmR^ seconded previous observations with *E. amylovora* ^WT^: no photo-antibacterial effect was observed at 1 μM, 10 μM and 100 μM PS concentration combined with 5 mM Na_2_EDTA even if doubling the radiant exposure to 53.2 J/cm^2^ (data not shown).

**Figure 4:**
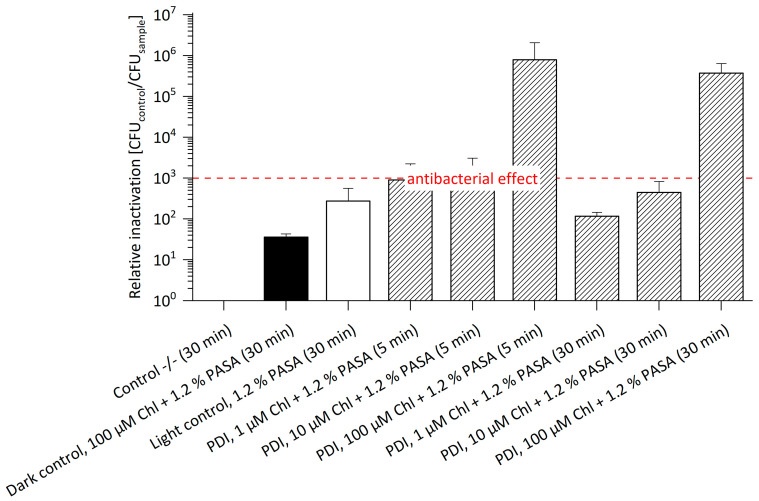
Relative Inactivation of *E. amylovora* ^SmR^ using Chl at 1, 10 or 100 µM and 1.2% PASA (*n* = 3) without Sm. *E. amylovora* ^SmR^ cultured in MPM without Sm could be photo-killed at 10 µM Chl and 1.2% PASA after 5 min incubation (3 log steps) and at 100 μM Chl and 1.2% PASA, with a relative inactivation of 5 log steps after 5 as well as 30 min. Incubation periods (in brackets) were 5 min or 30 min. Illumination was done at 395 nm and 26.6 J/cm^2^. CFU of Control -/- (mean): 8.0 × 10^7^.

Polyaspartic acid has a higher binding capacity of bivalent cations when compared to EDTA. Figure 4 illustrates the relative inactivation of *E. amylovora* ^SmR^ after PDI treatment with Chl at 1 μM, 10 μM or 100 μM concentration supplemented with 1.2% PASA (without Sm in media, see Table S3). The highest photo-killing efficacy was achieved at 100 μM Chl and 1.2% PASA, with a relative inactivation of 8.0 × 10^5^ after 5 min and of 3.8 × 10^5^ after 30 min incubation, respectively. An antibacterial effect could be shown at concentrations as low as 10 µM Chl and 1.2% PASA after 5 min incubation (3 log steps). Compared to photo-treatment of *E. amylovora* ^WT^ using Chl/PASA, a successful inactivation could only be shown at 100 µM Chl with 1.2% PASA and 30 min. incubation resulting in a reduction of 7 log steps [12]. The lower concentration of Chl required for an antibacterial effect against *E. amylovora* ^SmR^ when compared to *E. amylovora* ^WT^ is beneficial for field application, as this might allow for economic treatment. The somewhat higher photo-killing of the Sm-resistant strain at identical concentrations may be explained by the physiological modifications required for resistance, rendering cells more susceptible to PDI. However, the presence of 1.2% PASA might contribute to this effect, as both controls (dark control and light control) containing the additive show reduction in cell count of 1 log step in the dark and 2 log steps in the light control. These effects are tolerable as long as the plant tissues are not harmed. Evidence for this can be found in the recently published paper by Hamminger et al., which reports that this cell wall permeabilizing agents have no effect on the tissues of strawberry plants [13]. Therefore, the higher toxicity in the controls does not harm.

**Figure 5:**
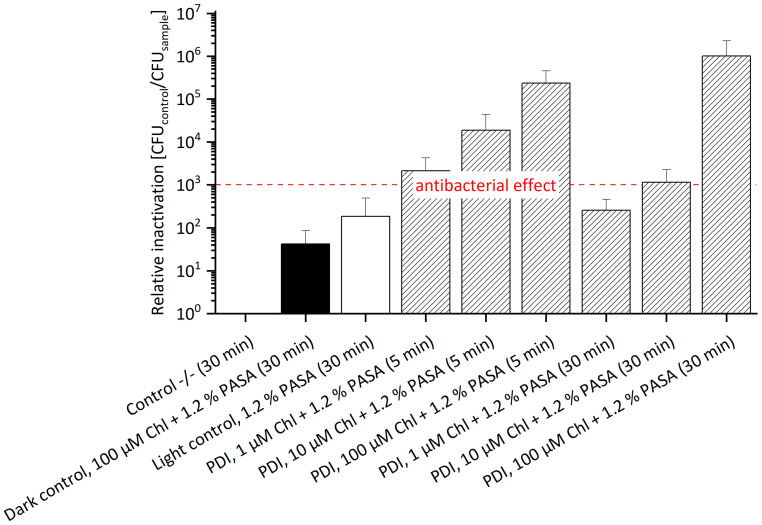
Relative Inactivation of *E. amylovora* ^SmR^ using Chl at 1, 10 or 100 µM and 1.2% PASA (*n* = 3) with Sm. The highest inactivation of *E. amylovora* ^SmR^ cultured in MPM with 100 µg/mL Sm of 1.0 × 10^6^ was achieved with 100 µM Chl and 1.2% PASA with an incubation of 30 min. An antibacterial effect was also observed at a concentration of 1 µM Chl and 1.2% PASA of 3 log steps, at 10 µM Chl and 1.2% PASA of 4 log steps, and at 100 µM and 1.2% PASA of 5 log steps, each with 5 min incubation. CFU of Control -/- (mean): 1.1 × 10^8^. Incubation periods were 5 min or 30 min (numbers in brackets) and illumination was done at 395 nm (26.6 J/cm^2^).

In complement, Chl with 1.2% PASA against *E. amylovora* ^SmR^ supplemented with 100 µM Sm was also tested in this experimental setting. An antibacterial effect was determined in almost all treatments (Figure 5, Table S4). Only the effect of 1 µM Chl with 1.2% PASA and 30 min incubation was not antibacterial. The highest antibacterial effect was observed with a relative inactivation of 1.01 × 10^6^ at 100 µM and 30 min incubation. It is remarkable that the relative inactivation at a concentration of 1 µM as well as 10 µM Chl combined with 1.2% PASA decreased with increasing incubation period, indicative of decay of the photoactive compound during incubation: at a concentration of 10 µM, the relative inactivation was 1.87 × 10^4^ after 5 min and 1.15 × 10^3^ after 30 min; at a concentration of 1 µM, the relative inactivation of 2.14 × 10^3^ decreased to 2.56 × 10^2^. Hence, photo-activation of Chl at lower concentrations led to a reduced efficacy of around 2 log steps with increased incubation period. This effect was not observed at high concentrations (100 µM). Chl is rather unstable and therefore undergoes degradation in the dark [38], which is more relevant at lower concentrations. The dark control contained Chl at 100 µM and 1.2% PASA and showed a value of relative inactivation of 4.20 × 10^1^ after 30 min incubation. The light control containing only 1.2% PASA showed a relative inactivation of 1.86 × 10^2^ after 30 min incubation, which is comparable to that of the treated sample of 1 µM Chl and 1.2% PASA and 30 min incubation. As already described above, the presence of Sm might have a slight additional effect.

When comparing the PDI efficacy of the two photoactive substances, B17-0024 and Chl, Chl induces an antibacterial effect at lower concentrations, at 10 µM Chl (w/o Sm), respectively 1 µM Chl (with Sm) after 5 min incubation. However, addition of PASA is required. Treatment with B17-0024 is antibacterial at 100 µM and 5 min incubation (5 log steps w/o Sm; 6 log steps with Sm) without additives.

In conclusion, due to its multimodal mechanism of action, PDI is able to kill bacteria irrespective of their resistance against antibiotics. For successful control of fire blight in orchards, the disease cycle must be considered. As for conventional treatments the control of blossom blight to inhibit propagation of the disease into plant tissues is mandatory. The maximal reduction of bacterial count induced by PDI in the laboratory setting was as high as seven orders of magnitude. Given the CFU count of controls of about 10^8^ cells/100 µL, a limited number of pathogenic bacteria does survive the treatment. However, the high initial CFU used in laboratory experiments is employed to achieve a high detection range and does not reflect the natural situation. Typically, in early stages of fire blight development, the number of pathogenic bacteria is significantly lower (hundreds to thousands, *pers. comm.* Prof. George Sundin, Michigan State University, USA) which reduces scattering of the activating light and might therefore allow for complete eradication of the disease.

## 3. Experimental Section

### 3.1. Bacterial Culture

All experiments were performed using the Sm-resistant strain of Gram (-) *E. amylovora* (*E. amylovora* ^SmR^), kindly provided by George Sundin, Michigan State University, USA. This strain was isolated in Washington, USA. New passages of bacteria were prepared with 100 µL (0.5%) of existing bacterial culture in 20 mL meat peptone medium (MPM) containing 5 g/L peptone (Sigma-Aldrich Chemie GmbH, St. Louis, MO, USA) and 3 g/L meat extract (beef extract, Carl Roth GmbH & Co. KG, Karlsruhe, Germany). Different concentrations of Sm (CAS 3810-74-0, Sigma-Aldrich) were added to the medium as required. The culture was then grown in aerobic conditions overnight (o/n) at 26 °C under constant agitation at 200 RPM in a shaking incubator (MaxQ 4000, Thermo Scientific, Marietta, OH, USA).

### 3.2. Growth Analysis of *Erwinia amylovora* ^SmR^

To test the impact of the Sm concentration on *E. amylovora* ^SmR^ growth curves were generated. A clear flat-bottom 96-well microplate (Greiner Bio-One International GmbH, Kremsmuenster, Austria) was used and each well was filled with a working volume of 200 µL, containing MPM amended with different concentrations of Sm in ascending order (w/o, 0.01, 0.1, 1 mg/mL Sm), and 0.5% o/n bacterial culture. Light scattering of bacterial solutions was determined by reading of absorption at 600 nm. Measurements were automatically performed for 10 h with a Spark multimode microplate reader (Tecan, Groedig, Austria). Instrument settings correspond to Tecan’s technical note [39]. Each sample was replicated 6 times. Mean values of these replicates were taken and corrected by blank to plot respective growth curves using MS Excel 2016. Control spectra of Sm showed no absorption of the antibiotic at 600 nm wavelength (data not shown).

### 3.3. Preparation of Stock Solutions

Sodium magnesium chlorophyllin (Chl, Carl Roth GmbH & Co. KG) and B17-0024 (kindly provided by Suncor AgroScience, Mississauga, ON, Canada) were dissolved in ultrapure water to prepare 10 mM stock solutions. They were stored at −20 °C in the dark until use. Polyaspartic acid (PASA; Baypure^®^DS100; Kurt Obermeier GmbH & Co. KG, Bad Berleburg, Germany) was acquired as 40% solution. A solution of 400 mM Na_2_EDTA (EDTA disodium salt dihydrate, VWR International, Leuven, Belgium) was dissolved in ultrapure water, adjusting the pH to 7.8. A stock of Sm at 100 mg/mL was prepared by diluting streptomycin sulfate salt powder (CAS 3810-74-0, Sigma-Aldrich) with ultrapure water. Aliquots were stored at −20 °C.

### 3.4. Photodynamic Inactivation of Erwinia amylovora

For the in vitro PDI experiments, bacterial suspensions were centrifuged at 830 RCF for 3 min (Centrifuge 5417R, Eppendorf, Hamburg, Germany). After carefully removing the supernatant, the pellet was resuspended in Dulbecco’s modified Phosphate Buffered Saline (DPBS, Sigma-Aldrich Chemie GmbH) containing either B17-0024 or Chl (1 μM, 10 μM and 100 μM). Chlorophyllin was supplemented with 5 mM Na_2_EDTA or 1.2% PASA for cell wall permeabilization. Triplets of 500 µL of each sample were transferred to 24-well microplates and incubated for 5 or 30 min under constant agitation (Flow Laboratories, DSG Titertek, microplate shaker) in the dark. Illumination followed straight after the incubation period from below using a LED-array consisting of 480 LEDs (diode type L-7113UVC, Kingbright Electronic Europe GmbH, Issum Germany) with a dominant wavelength of 395 nm. Given the radiant exposure of 26.6 J/cm^2^, the exposure time was 15:50 min. During illumination samples were permanently agitated to avoid sedimentation of bacteria. The double negative control (Control -/-) contained no PS and the sample was not illuminated. The dark control was incubated with the highest concentration of PS used in the experiment, but the sample was not exposed to light. The third control was the light control, without PS in the sample but exposed to light. Controls were otherwise treated identical to all other samples. After illumination, all samples were serially diluted in DPBS up to a dilution factor of 10^−7^ and 50 µL of each prepared dilution were plated on petri dishes containing MPM and 1.5% Agar-Agar (Kobe I, Carl Roth GmbH & Co. KG), and if necessary 100 µg/mL Sm. The plates were incubated for 48 h at a constant temperature of 26 °C in the dark (Heraeus incubator Typ B5042 E, Hanau, Germany). Every set of experiments consisted of 3 independent biological replicates.

### 3.5. Data Analysis

For each treated and untreated sample, viable cells recorded as colony-forming units (CFU) were counted and multiplied by 2 to determine the number of CFU per 100 µL. For calculation of the relative inactivation the CFU of the double negative control was divided by the CFU of the sample for each biological replicate [40]. In the case that no CFU were visible (= detection limit), the CFU of the double negative control was divided by 1. Mean value (mean) and standard deviation (SD) were calculated for all replicates. According to the standards of the American Society for microbiology an antibacterial effect will be claimed if cell killing exceeds 3 orders of magnitude (removal of 99.9%) [41,42]. A red dashed line was added to the graphs to illustrate this antibacterial effect. All data of experiments presented in this study can be found in Tables S1–S4 in the supplementary part.

## 4. Conclusions

This is the first study using PDI against plant pathogens resistant to antibiotics. PDI treatment can successfully kill bacteria of the Sm-resistant strain of *E. amylovora* ^SmR^. In comparative testing of the synthetic B17-0024 and the naturally derived Chl, the highest photo-efficiency was shown with B17-0024 at a concentration of 100 µM. Nevertheless, promising results were also obtained with Chl in combination with 1.2% PASA. Here, antibacterial effects against *E. amylovora* ^SmR^ were already triggered with 10 µM Chl and 1.2% PASA. The presence or absence of Sm in the o/n bacterial culture or during PDI seems to play a role in the degree of PDI efficacy. Addition of Sm results in a slight increase of overall toxicity towards *E. amylovora* ^SmR^. Keeping in mind that neither the photoactive compounds, nor the additives influenced the growth and development of strawberry plants [13], PDI based on natural substances or derivatives thereof shall allow for eco-friendly and sparing treatment of bacterial-induced plant diseases.

## Data Availability

Not applicable.

## Supplementary Materials

The following supporting information can be downloaded at: www.mdpi.com/article/10.3390/antibiotics11050544/s1, Table S1: PDI of *E. amylovora* ^SmR^ using B17-0024 w/o Sm in medium (*n* = 3); Table S2: PDI of *E. amylovora* ^SmR^ using B17-0024 with 100 µg/mL Sm (*n* = 3); Table S3: PDI of *E. amylovora* ^SmR^ based on Chl supplemented with 1.2% PASA w/o Sm in medium (*n* = 3); Table S4: PDI of *E. amylovora* ^SmR^ based on Chl supplemented with 1.2% PASA and 100 µg/mL Sm (*n* = 3).
