# Supplementary material for: Breaking the Rebellion: Photodynamic Inactivation against Erwinia amylovora Resistant to Streptomycin
Source: Antibiotics (Basel). 2022 Apr 19;11(5):544. doi: 10.3390/antibiotics11050544 (PMC9137749; doi:10.3390/antibiotics11050544)
Supplement: Supplementary file 1 [file antibiotics-11-00544-s001.zip › antibiotics-1675745-supplementary-done.pdf]

antibiotics

MDPI

Supplementary

Table S1: PDI of *E. amylovora* ^{SmR} using B17-0024 w/o Sm in medium (n = 3)

	CFU / 100 µL			relative inactivation [$CFU_{control}/CFU_{sample}$]		
	mean	±	SD	mean	±	SD
Control -/- (30 min)	4.57E+07	±	1.15E+07	1.00E+00	±	0.00E+00
Dark control, 100 µM (30 min)	5.11E+07	±	1.61E+07	9.23E-01	±	1.99E-01
Light control (30 min)	5.15E+07	±	2.06E+07	1.01E+00	±	5.93E-01
PDI, B17-0024, 1 µM (5 min)	6.80E+07	±	1.08E+07	6.76E-01	±	1.74E-01
PDI, B17-0024, 10 µM (5 min)	4.28E+07	±	2.35E+07	1.25E+00	±	6.23E-01
PDI, B17-0024, 100 µM (5 min)	3.32E+03	±	3.02E+03	2.44E+05	±	4.03E+05
PDI, B17-0024, 1 µM (30 min)	5.46E+07	±	9.04E+06	8.52E-01	±	2.70E-01
PDI, B17-0024, 10 µM (30 min)	1.67E+07	±	1.42E+07	8.08E+00	±	1.00E+01
PDI, B17-0024, 100 µM (30 min)	5.53E+01	±	4.71E+01	1.17E+07	±	1.93E+07

Table S2: PDI of *E. amylovora* ^{SmR} using B17-0024 with 100 µg/ml Sm (n = 3)

	CFU / 100 µL			relative inactivation [$CFU_{control}/CFU_{sample}$]		
	mean	±	SD	mean	±	SD
Control -/- (30 min)	7.11E+07	±	3.11E+07	1.00E+00	±	0.00E+00
Dark control, 100 µM (30 min)	1.13E+08	±	1.95E+07	6.61E-01	±	3.91E-01
Light control (30 min)	8.07E+07	±	3.75E+07	9.42E-01	±	3.13E-01
PDI, B17-0024, 1 µM (5 min)	8.33E+07	±	4.56E+07	9.71E-01	±	3.77E-01
PDI, B17-0024, 10 µM (5 min)	4.93E+07	±	1.62E+07	1.56E+00	±	9.58E-01
PDI, B17-0024, 100 µM (5 min)	1.63E+01	±	9.71E+00	5.79E+06	±	3.69E+06
PDI, B17-0024, 1 µM (30 min)	6.97E+07	±	3.70E+07	1.15E+00	±	4.09E-01
PDI, B17-0024, 10 µM (30 min)	3.56E+06	±	1.74E+06	2.14E+01	±	5.93E+00
PDI, B17-0024, 100 µM (30 min)	3.38E+02	±	5.82E+02	3.82E+07	±	5.72E+07

Table S3: PDI of *E. amylovora* ^{SmR} based on Chl supplemented with 1.2% PASA w/o Sm in medium (n = 3)

	CFU / 100 µL			relative inactivation [$CFU_{control}/CFU_{sample}$]		
	mean	±	SD	mean	±	SD
Control -/-	7.96E+07	±	2.68E+07	1.00E+00	±	0.00E+00
Dark control, 100 µM Chl + 1.2 % PASA (30 min)	2.23E+06	±	7.58E+05	3.62E+01	±	7.13E+00
Light control, 1.2 % PASA (30 min)	7.23E+05	±	7.72E+05	2.76E+02	±	2.91E+02
PDI, 1 µM Chl + 1.2 % PASA (5 min)	8.41E+05	±	1.20E+06	9.04E+02	±	1.35E+03
PDI, 10 µM Chl + 1.2 % PASA (5 min)	1.34E+05	±	9.77E+04	1.41E+03	±	1.67E+03
PDI, 100 µM Chl + 1.2 % PASA (5 min)	1.45E+03	±	1.92E+03	7.98E+05	±	1.28E+06
PDI, 1 µM Chl + 1.2 % PASA (30 min)	7.32E+05	±	4.05E+05	1.17E+02	±	2.77E+01
PDI, 10 µM Chl + 1.2 % PASA (30 min)	2.95E+05	±	2.14E+05	4.50E+02	±	3.77E+02
PDI, 100 µM Chl + 1.2 % PASA (30 min)	1.65E+02	±	1.51E+02	3.76E+05	±	2.63E+05

Table S4: PDI of *E. amylovora* ^{SmR} based on Chl supplemented with 1.2% PASA and 100 µg/ml Sm (n = 3)

	CFU / 100 µL			relative inactivation [$CFU_{control}/CFU_{sample}$]		
	mean	±	SD	mean	±	SD
Control -/-	1.13E+08	±	1.27E+07	1.00E+00	±	0.00E+00
Dark control, 100 µM Chl + 1.2 % PASA (30 min)	1.25E+07	±	1.81E+07	4.20E+01	±	4.31E+01
Light control, 1.2 % PASA (30 min)	9.38E+06	±	8.26E+06	1.86E+02	±	3.09E+02
PDI, 1 µM Chl + 1.2 % PASA (5 min)	2.43E+06	±	4.13E+06	2.14E+03	±	2.12E+03
PDI, 10 µM Chl + 1.2 % PASA (5 min)	1.07E+06	±	1.83E+06	1.87E+04	±	2.54E+04
PDI, 100 µM Chl + 1.2 % PASA (5 min)	2.67E+05	±	4.62E+05	2.35E+05	±	2.18E+05
PDI, 1 µM Chl + 1.2 % PASA (30 min)	1.91E+06	±	2.76E+06	2.56E+02	±	2.06E+02
PDI, 10 µM Chl + 1.2 % PASA (30 min)	4.16E+06	±	7.08E+06	1.15E+03	±	1.12E+03
PDI, 100 µM Chl + 1.2 % PASA (30 min)	6.04E+04	±	1.04E+05	1.01E+06	±	1.27E+06

Abbreviations: ***CFU***, *Colony forming unit(s);* ***Chl***, Sodium magnesium chlorophyllin *(Na-Chlorophyllin);* ***DPBS***, *Dulbecco's modified phosphate buffered saline;* ***E. amylovora*** ^{SmR}, *Streptomycin resistant strain of Erwinia amylovora;* ***E. amylovora*** ^{WT}, *Susceptible strain of Erwinia amylovora (type strain, DSM 30165);* ***MIC***, *Minimum inhibitory concentration;* ***mean***, *Mean value;* ***MPM***, *Nutrient medium with meat extract and peptone;* ***Na_2EDTA***, Ethylenediaminetetraacetic acid disodium salt dihydrate; ***o/n***, *Overnight;* ***PASA***, *Polyaspartic acid;* ***PDI***, *Photodynamic Inactivation of microorganisms;* ***PS***, *Photosensitizer;* ***RCF***, Relative centrifugal force; ***ROS***, *Reactive oxygen species;* ***RPM***, *Revolutions per minute of rotor;* ***SD***, *Standard deviation;* ***Sm***, *Streptomycin sulfate salt;* ***w/o***, *Without*
